# Are we Genomic Mosaics? Variations of the Genome of Somatic Cells can Contribute to Diversify our Phenotypes

**DOI:** 10.2174/138920210793175949

**Published:** 2010-09

**Authors:** P.A. Astolfi, F. Salamini, V. Sgaramella

**Affiliations:** 1 Department of Genetics and Microbiology, University of Pavia, Italy; 2 PTP, Lodi, Italy

**Keywords:** Copy number variation, aneuploidy, genomics, epigenomics, transposons, retroposons.

## Abstract

Theoretical and experimental evidences support the hypothesis that the genomes and the epigenomes may be different in the somatic cells of complex organisms. In the genome, the differences range from single base substitutions to chromosome number; in the epigenome, they entail multiple postsynthetic modifications of the chromatin. Somatic genome variations (SGV) may accumulate during development in response both to genetic programs, which may differ from tissue to tissue, and to environmental stimuli, which are often undetected and generally irreproducible. SGV may jeopardize physiological cellular functions, but also create novel coding and regulatory sequences, to be exposed to intraorganismal Darwinian selection. Genomes acknowledged as comparatively poor in genes, such as humans’, could thus increase their pristine informational endowment. A better understanding of SGV will contribute to basic issues such as the “nature *vs* nurture” dualism and the inheritance of acquired characters. On the applied side, they may explain the low yield of cloning *via* somatic cell nuclear transfer, provide clues to some of the problems associated with transdifferentiation, and interfere with individual DNA analysis. SGV may be unique in the different cells types and in the different developmental stages, and thus explain the several hundred gaps persisting in the human genomes “completed” so far. They may compound the variations associated to our epigenomes and make of each of us an “(epi)genomic” mosaic. An ensuing paradigm is the possibility that a single genome (the ephemeral one assembled at fertilization) has the capacity to generate several different brains in response to different environments.

“Where will genomics be ten years from now? As sequencing capacity increases globally and the data quality improves, we will move beyond the current goal of one genome per person by sequencing multiple genomes per person… The genomic revolution is just beginning.” (J. C. Venter, Nature 464:676-677, 2010).

## INTRODUCTION

It is known that the famous physicist Erwin Schrödinger, Nobelist in 1933 “for the discovery of new productive forms of atomic theory”, pioneered the advent of molecular biology with a widely quoted booklet [1] which stimulated many colleagues of different expertise to converge on the study of DNA. Less known is a statement where Schrödinger serendipitously anticipated a violation of a tenet to be formulated after the unveiling of the DNA double helix. In particular Schrödinger stated: “…*all the cells of our body are exactly identical for what pertains their chromosomes*”*, *and in a footnote added: “*But biologists will forgive me if I do not mention the exceptional cases of mosaics*”. It is probable that the “mosaics” alluded to here were the products of intrauterine exchanges of embryonic cells between fraternal twins. 

The tenet at risk of violation descended directly from the “central dogma of molecular biology”, promulgated in the early 1950s by Francis Crick, codiscoverer of the double helix and admittedly one of the many physicists converted to molecular biology by Schrödinger. Crick’s dogma prescribed that in living cells the genetic information flows from DNA to RNA and hence to proteins, but not backwards; in the 1970s it had to be revised because of the discovery of reverse transcription of RNA into DNA. But by and large the dogma still stands as a cornerstone of the biosciences, especially with regard to the logically consequent invariability (a.k.a. continuity or stability) of the somatic genomes. A significant statement has appeared recently in a Nature commentary [2]: “*Whereas the hard-wired genome is virtually identical in all of a person's roughly 250 different types of tissue and is essentially stable during that person's lifetime, the epigenome changes during development such that each cell type has its own characteristic set of marks. These marks change with age and may also change, possibly in a heritable way, in response to environmental stress*”*.* 

It is also known that epigenomics studies the complex but reversible modifications of chromatin (*i. e.*, DNA plus the wealth of accompanying proteins and non-coding small RNA) [3], whereas genomics addresses the base sequences of the DNA present in the living cells. But if it is questionable that epigenomic marks are *“directly dependent”* on DNA sequence, as Ptashne *et al.* state in a recent note to Nature [4], the two are not unrelated: *“In contrast to the DNA sequence which is identical in all tissues, the patterns of chromatin modifications and DNA methylation are tissue specific. Thus our genome contains two layers of information: the DNA sequence inherited by our parents which is fixed throughout life and identical to most extent in all the cells and tissues of our body, and chromatin and methylation patterns which are cell and tissue specific. It is clear that gene function and thus the phenotype could be influenced not only by the gene sequence but also by the epigenetic programming of the sequence.”* [5]. Even more explicitly it has been stated that: *“The proper development of multicellular organisms entails the distinct specification of disparate cell types. Despite having identical genomic sequences, different cell types exhibit substantially different profiles of gene expression, and their cellular identity must be conserved during somatic cell divisions. How then are cell-specific gene-expression patterns specified and maintained? It is now recognized that the key is ‘epigenetics’: the stable and heritable information that is distinct from DNA sequences and fostered by specialized mechanisms.” *[6]. 

We propose here that there is a third layer of information, represented by the variations affecting the somatic genomes throughout development: the ensuing genomic mosaicism responds not only to epigenetic fluctuations often traceable to the environment [5-8], but also to genetic determinism, hence to both chance and necessity. For the transactions affecting DNA sequences we suggest the expression Somatic Genome Variations (or SGV), as preferable to the common Copy Number Variations (CNV), given that, *e. g.,* non-replicative transpositions and inversions are not *stricto sensu* CNV.

At present SGV are perceived no longer as syndromic to disease, but also as marks of differentiation. The acceptance of their existence, and even of their importance, has gone through the stepwise and haphazard additions of bits of evidence, but is steadily consolidating. The picture has been aptly summarized as follows: *“Fortunately, in the face of ever-increasing level of variation being discovered in the human genome, there is at least one touchstone: the genomes of all the cells in a cancer-free individual are the same. However, even in this bedrock, a few cracks have appeared. For example, it is well-known that immune cells undergo rearrangements and deletions at immunoglobulin loci. Cases are also known of genomic mosaicism, even as far as aneuploidy in sub-population of cells […]. But, aside from these peculiar exceptions, and barring mutations accumulated as part of the ageing process, the genomes in all of your cells are identical. Or are they?” *[9]. In order to help answering this rhetorical question we must critically review the main notions supporting the invariability of the somatic genome: they include the extraordinary accuracy recognized to *in vitro* DNA replication, the stability of DNA borne by somatic and germinal nuclei, the constancy of the 2:1 ratio of their respective masses, the persistence of familiar traits through different generations. The seal to these notions was provided in the early 1960s by cloning *via* somatic cell nuclear transfer (SCNT) in amphibians: their main finding was that the somatic nuclei transferred into enucleated unfertilized or fertilized oocytes could produce healthy organisms; their main conclusion was that such nuclei should be considered totipotent. But those experiments had at least three caveats: (a) only embryonic and fetal cells would provide functional nuclei; those from adult cells would fail, at least in those early days; (b) yields have since remained close to  1% of transferred nuclei, essentially regardless of their donor cells; (c) the resulting totipotence does not necessarily imply full identity of the involved genomes, as shown by the few clones grown to adulthood, which were taken as genotypically “identical” to the nuclei donors, but resulted phenotypically different, at least health-wise. 

But the rhetorical question raised above by Dear [9] cogently underscores the complexities of the SGV issue. The debate over the invariability of the somatic genome is more than a century old: for a review see, *e. g.*, [10]. At the beginning it was thought that multicellular organisms would develop their manifold traits either through a differentially regulated expression of a constant genome (Delage, Spemann and others), or alternatively through genomic structural variations which would lead to different patterns of gene expression (Driesch, Weismann and others). Mutations were thought to occur in single cells, where they would disrupt gene structure and function, and thus occasionally trigger dramatic genomic alterations, uncontrolled growth of clonal progeny cells into anomalous and eventually diseased tissues  [11]. Shortly, the notion of the variation of the somatic genome could be accepted, as long as it was associated to pathology. That under normal conditions all the somatic cells of an organism would harbor the same genome had become almost a second dogma: it predicated its invariability in the somatic cells of any complex organism throughout, and in spite of, its differentiation and development. But its tenet had to coexist with some contrasting evidences: 

(a) in living cells the genetic information flows also from RNA back to DNA, and does so both spatially (*i.e.,* in different cells), and temporally (*i.e.,* at different developmental stages): the apostle of this tenet was Howard Temin, who studied RNA viruses and stated that *“This normal process of information transfer in cells could not exist only for its ability to give rise to viruses. It must exist as a result of its role in normal cellular processes, for example cell differentiation, antibody formation and memory” *[12]; remarkable prediction indeed, especially for what concerns memory and more in general cognitive functions. But still not enough to secure an official *imprimatur* to Temin’s doctrine even long after the subsequent extensive characterization of retroviruses [13] and in general of retroelements [14]; 

(b) the eukaryotic genomes host a variety of transposable elements (TE), at least in part responsible for improving the response of the organisms to the environment; the apostle of this tenet was Barbara McClintock, who clearly summarized her ideas as follows: “*I believe that there is little reason to question the presence of innate systems that are able to restructure a genome. It is now necessary to learn of these systems and to determine why many of them are quiescent and remain so over long periods of time, only to be triggered into action by forms of stress, the consequences of which vary according to the nature of the challenges to be met. In addition to modifying gene action, these elements can restructure the genome at various levels, from small changes, involving a few nucleotides, to gross modifications, involving large segments of chromosomes, duplications, deficiencies, inversions and other more complex reorganizations.*” [15] Both Temin and McClintock were eventually awarded fully deserved Nobel Prizes, but their colleagues are still taking a while to accept their teachings, and hesitate to recognize that the integration of DNA copies of RNA sequences into the nuclear genome, as well as the chromosomal vagaries of TE, are incompatible with the stability of the somatic genomes and must lead to their restructuring. 

Probably easier to be accepted are the concepts put forward by Zeh *et al.* [16] with regard to the causative relationship of epigenetics with regard to transpositions especially in evolution: *“According to the “epi-transposon hypothesis”, physiological stress, associated with major climatic change or invasion of new habitats, disrupt epigenetic silencing, resulting in TE reactivation, increased TE expression and/or germ-line infection by exogenous retroviruses. Mobilized TE rapidly restructure the genome and alter gene expression patterns…”. *However their correlations to development are steadily emerging, as revealed by the following quotations from Kazazian and Ostertag [17]: “*Time and further research will tell whether McClintock’s hypothesis that TE have a significant role in an organism’s development can be extended from maize to humans, and specifically to the functions of human neurons.*”. Discussing nervous system disorders, Lee & Lupski [18] display a more open view and hypothesize that *“gene copy number variations due to structural alteration may be responsible for both normal and abnormal behavioral phenotypes”*.

Many other instances of intraindividual somatic genome variations can be predicted at the theoretical level; several have indeed been described and they do not necessarily violate consolidated enunciations, since they act through recombination and similar notorious and legitimate transactions. But apart from mechanisms, their impact on the concept that all our cells harbour the same genome may threaten the central dogma and question some extant major projects.

## STATE OF THE ART

It is still accepted that at each cellular division the structure of the genome as assembled at fertilization is faithfully duplicated together with the rest of the cell [6], apart from epigenetic changes and occasional ploidy variations [19]. In this way the genetic information is transmitted essentially unchanged to all the cells of our developing body, in its germinal as well as somatic lineages. Thus the phenotypes of the organism were seen as the gradual decoding of the information carried by a constant genome and expressed in the many features proper of our ~250 kinds of tissues, with all the subroutines of sophisticated programs of gene activation/inactivation. In cells where changes are the rule, one notable exception would therefore stand out: the genome, admittedly a fundamental element of our soma. Thus, throughout development the genome would remain unchanged. But several documented objections are raised against this view: they provide support to frequent, different and substantial manifestations of SGV, which go from single base substitutions or modifications to chromosome-scale events and may differ from traditional mutations since they are at least partly programmed. Some of the relevant objections to the invariability of the genome stem out of theoretical considerations: take, *e. g.,* the genome huge size (in terms of bp man has ~6.10^9^) which makes it an easy target for damages (~10^4^ per cell per day according to Jackson & Bartek [20]) , the number of cells contributing to the steady state of a human organism (~10^14^), the high number of mitoses (~10^16^) necessary for the organism’s full maturation and susceptible to errors and mishaps. Apart from these quantitative data, in the genome we have an extraordinary abundance of all sorts of transposable/unstable sequences, often exceeding 50% of the total DNA [16], and a reasonable confidence that most of these considerations apply to both animals [21] and plants [22]. Finally, there is the paradox of the low number of the human coding genes, 22,287 as acknowledged in 2006 by the Ensemble 34rd Catalogue of the IHGSC, as such close to those of a nematode [23] and to one third of a protist [24]; it cannot be excluded that such a gene paucity could be partially compensated through the intervention of SGV. 

The strength of all these arguments contrasts with the weakness of the extant experimental data: beyond the classical cases of the gene reorganization controlling the immune response in vertebrates, of the macro/micro-nuclei transactions in protozoa, and of the telomere attritions punctuating the development of most eukaryotes, reports detailing tissue-specific and age-related variations in the genomes of eukaryotic cells are preciously few [24, 25]. 

Many recent data are, however, coalescing and paving the way to a desirable and feasible experimental characterization of SGV. One is the reported low success rate of cloning *via* SCNT, which is more compatible with rearrangements suffered by the relevant somatic cells genomes [26], than with technical hurdles [27]. Suggestive indications can be excerpted from recent genomic data: thus*, *the similarity of the estimated numbers of SGV in a given genome and of the gaps persisting in the sequences of the “quasi-completed” genomes available thus far [28, 29] point to an involvement of SGV. When the genomes of a mixture of cells of the same lineage of the same individual are being sequenced, at the SGV sites their genomes are likely to host heterogeneous sequences typical, or even unique, of each cell under study. It is in fact possible that some cells could bear alterations which result from untemplated, error-prone DNA repair at the sites of their occurrence, as in the assembly of the immunoglobulins and related genes. The genomic revolution [30], the unpredicted discoveries of systems similar to myelomas and the zooming on some of the most obvious of such sites will help to clarify the issue in the future. Advancements are expected from the increasingly powerful “next generation” sequencing tools. Discussing the interesting discovery that the jawless sea lamprey *Petromyzon marinus* looses close to 20% of their somatic genome during development, Eichler and colleagues underline the programmed nature of their loss which includes at least one transcribed gene, and conclude: “*Thus, the loss of DNA could result in the generation of coding, promoter, enhancer, etc. sequences that impart unique functionality to somatic cell lineages.*” [31].

As to their insurgence mechanisms, SGV may be triggered by genetic programs responding also to external stimuli/stresses [8, 32, 33], DNA replication/repair errors [34], exposure to variably processed reverse transcripts of viral genomes [35] and of pseudogenes [36], horizontal gene transfers [37], etc. In some cases the environmental circumstances may elicit a programmed cellular death (apoptosis). Alternatively, the somatic genomes may adapt to these events *via* gene copy number variations, TE repositioning and representation, expansion or contraction of repeats (long or short, tandem or dispersed), chromosomal aberrations. Ultimate measures may be the loss of entire chromosomes and eventually the elimination of the whole nucleus, as in mammalian erythrocytes. All, *nota bene*, under the aegis of a rigorous somatic Darwinian selection. 

Within the same organism different cell lineages, and possibly within the same lineages, single cells may display peculiar instances of SGV. Remarkably, cleavage stage human embryos are reported to suffer sudden dramatic insurgence of severe complex chromosomal aberrations, admittedly absent in the participating gametes [38, 39; see also the accompanying contribution of Robberecht et al. in this issue] and eventually responsible of undetected abortions of zygote-derived structures [40]; later on, the emerging germ lines may enjoy a peculiar protection against the insurgence of SGV as compared to the rest of the soma. Conversely, a preferential susceptibility to SGV may be found associated with cells positioned next to the boundary between environment and organism and progressing to a postmitotic stage, such as functional neurons [41, 42]. It has been proposed that the ensuing DNA rearrangements tend to occur close to the chromosomal sites involved in segmental duplications and to act as hotspots for further rearrangements and recombinations [43]. The genomic alterations caused by SGV may involve up to ~20% of the human genome [44]. This may affect many functions and support an inordinately increase in their frequency and size throughout development and aging [45]. An important message emerging from these data is that SGV may feature in supposedly “normal” cells and not only represent symptoms of extant or incumbent disease, from cancer to severe neurodegenerative disorders like Alzheimer’s disease and ataxia telangiectasia [46]. This last consideration gains credibility thanks to the blurred demarcation between physiology and pathology, ostensibly occurring in derangements of the immune system [47] and, probably, of the nervous system [40].

**Fig. (1):**
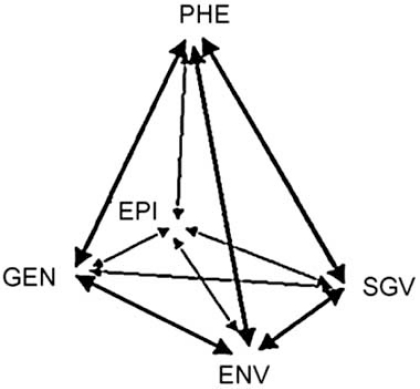
The “ontogenic pyramid”. The phenotype (PHE) may be seen as the result of four variables: the original zygotic genome (GEN), the environments (ENV), the epigenome (EPI) and the somatic genome variations (SGV). The two headed arrows are meant to suggest complex reciprocal interactions between them. From Sgaramella & Astolfi [39], with permission.

By altering the organization of the genome of affected cells, and hence their coding capacity as well as their regulation, SGV may account for a good share of the organismal phenotypic differences. This hypothesis is becoming attractive and in the last few years SGV have been enlisted within the traditional elements concurring to the shaping of the phenotype: these include the zygotic genome, as assembled at fertilization; the environment, in its macro-components, surrounding the whole organism as it develops, as well as its micro-components, surrounding each cell; and the epigenome, *i. e*., the sum of the transient, inducible and partly inheritable modifications of the chromatin of the cells. These concepts have been elaborated in a graphical representation for which the term “ontogenic pyramid” has been proposed (Fig. **1**), to suggest an idea and a model that in due course will probably undergo stringent criticism and re-elaboration. 

A productive experimental effort aimed to the characterization of SGV must first detail out a broad strategic plan and then an accurate description of the operations in term of materials and methods. The optimal experimental organism(s) should display several useful traits, such as a complex developmental program leading to lineages as differentiated as possible; representative cells should derive from tissues originating from each of the three layers of an animal gastrula or from plant embryo structures; cells easy to be isolated as pure and uncontaminated as possible would be preferable; a reliable “reference” genome should be available for comparing the changes emerging during development. A reasonable prediction as to which sequences may be more amenable to SGV could first consider (retro)transposons, then elements such as LINEs and SINEs, especially those sequences known or suspected to assume anomalous non-B conformations [44, 48]. It has been reported that, in the spectrum of the events responsible for human genome variations, deletions are more frequent than insertions or other rearrangements [49]: this is reasonable and may reflect a compensatory removal of sequences following synthetic (retro)transpositions otherwise responsible of an unbearable overgrowth of the genome size [50]. Relevant to this is the observation that during development: *“The set of genes deleted is not random in the (human) genome; there is a clear association with segmental duplications among larger deletion events. In addition to previously reported associations with immunity, defence, chemosensation and drug detoxification, other functional gene categories emerge in these studies, such as signal transduction, sex hormone metabolism and cancer susceptibility.”* [51]. Somehow ironically, indications for cells more susceptible to SGV may turn out to be a major contribution to science of the once much hyped organismal cloning: the finding that animal tissues (*e. g.,* neurons) are more recalcitrant than others (*e. g.*, cumulus) at producing mouse clones *via* SCNT could betray some tissue specificity of SGV [26, 52, 53] rather than technical hurdles. A second criterion for spotting cells more prone to SGV and thus more useful for their characterization, could be the extent of their epigenetic modifications: these changes control the structure of chromatin, and hence possibly vary the sequence of its DNA. This may occur through transcription, then reverse transcription of some resulting RNA followed by the genomic integrations of their retrotranscripts as retroposons and pseudogenes [5, 16, 35, 54].

In a concerted approach to the study of SGV particular attention may go to: (1) their manifold nature, (2) their time-course during growth, (3) their molecular mechanisms, (4) their significance *vis-à-vis* the physiological as well as the pathological development of the organism, (5) their relevance on applied science as it regards, *e. g.,* reproductive and regenerative cloning, induction of pluripotency, DNA analysis, etc.

It would be desirable to focus on plant and animal models, better if endowed with relatively short life spans: this would facilitate the monitoring of the time course of SGV from early developmental stages to senescence and death. Among plants, a suitable system may be represented by *A. thaliana* [55], whereas among animals mice may be preferable, in spite of the somehow lower fraction of repeats in their genome [56]. Needless to say that humans are the ultimate objective.

From a practical viewpoint, it would be proper to study SGV in as few cells as possible, given the peculiar, if not the unique, nature of SGV. Single cell genome analysis would be highly desirable and may be reached in a not too distant future [57].

The study of SGV rests heavily on the use of efficient sequencing technologies [26], to be applied to whole genomes, or to parts selected because of their higher predisposition to SGV, as based either on prior knowledge, *e. g.*, retroposons, as discussed by Sen *et al. *[58], or on acquired experimental evidence (*e. g.,* gene duplication sites), as discussed by Rohlin *et al.* [30], and by many others [59-62]. Possible concomitant epigenetic changes are expected to augment the complexities of the experimental approaches to SGV characterization.

In a note appeared almost five years after the first “completion” of the Human Genome Project it was remarked that “*Uncovering the genetic basis of human phenotypic differences requires a comprehensive understanding of all forms of genetic variation*”, and the following explicit conclusions were reached: “*First, no single optimized approach has been developed yet to systematically capture all structural variation in the human genome. Second, it is likely that several thousand additional common structural variants await discovery. Their abundance, enrichment in environmental-interaction genes and their apparent attributes in terms of natural selection suggest that these genetic lesions will be important in genetic disease. A more systematic, unbiased approach to discover and genotype such variation is required. A Human Genome Structural Variation Project dedicated to the characterization of not only deletions but also of insertions and inversions should become a priority for the human genetics and human genome sequencing communities in an effort to further widen the spectrum of human genetic variation*” [51]. The relevance of these remarks has only been increasing in these last few years. If we were to place the adjective “somatic” before the substantives “genetics” and “genome”, as they recur in Eichler’s note, and to use the prefix “(epi-)” ahead of both of them, and if we were to enlarge the scope of his exhortations beyond disease to normal human traits, we would find a close match to the subject and the scope of this paper. 

## CONCLUSIONS

In shaping the physical, behavioural and cognitive human phenotypes, nature and nurture cooperate, probably not as independent entities. Rather they interact in a complex manner: accordingly, the genotype, the environmental stresses and/or stimuli, the epigenome [8] and finally SGV talk to each other so that eventually the genomes of any somatic cells may end up as unique patchworks and as such differ not only from the ephemeral genome of the zygote, but also among its many tissutal or even cellular versions [7, 43]. 

In a sense we are what we are because of our genes, but our genes are what they are because their roles are played in different macro- and micro-environments. Both the genes we inherit and the environments we are placed in result from random samplings out of countless alternatives. The dialogue between genes and environment dictates the necessity of our genetic/genomic mosaicism, its augmentation during development, its derangement during disease, possibly of its exaltation in forging our mind. Such interactions remind some of the ideas Lamarck published in 1809, the same year of Darwin's birth [63]. This underscores the desirability of an analysis of the genome and the epigenome of different cell lineages at different developmental stages, and, possibly, at the single cell level, if we want to understand reliably the biologic determination of what we are genomically and epigenomically at any given point of our life. In this regard, somehow similarly to their colleagues physicists who in the last century integrated Heisenberg’s “indetermination principle” in the characterization of the nuclear elementary particles, today geneticists may be advised to consider an “indeterminability” of the genotype-phenotype relationship, given the ever-changing nature of both partners [23, 43, 64]. If all this is confirmed, when facing the ancient dilemma concerning the interactions of nature *vs* nurture in shaping our traits, we may find that the limits of the definition of the multifarious contributions of nurture (*i. e.,* diet, education, conscious and subconscious stresses and/or stimuli, etc) are no less severe than the contributions of nature (*i. e.*, our many possible genomes), as recently discussed by several authors [32, 33, 65]. The challenge becomes daunting when we envisage a role of SGV in shaping our neurons to produce just one brain (ours) out of the many possible ones which our inherited genome could have borne out by interacting with its unarguably changing environments: *“If correct, this hypothesis predicts that individual neural cells will, in fact, have distinctive spatially and temporally defined genomic sequences and chromatin structure.” *[66]. Nothing new under the sun: this prediction simply adds more appeal to the teachings untiringly dispensed over the years by Temin [12], McClintock [15] and Edelman [67], and will perhaps stimulate some re-evaluation of the significance of the notion that *“brain plasticity, while considerable, is constrained by genetics.”* [68]. 

The increasing overlapping of the broad fields of genomics and epigenomics emphasizes the importance of a large-scale approach to the study of the development-related variations of chromatin (the cellular structure which 140 years ago was dubbed “nuclein” by Miescher and launched molecular genetics), as well as of their phenotypic and behavioural consequences. The size of the endeavours and the expected value of the results recommend an integrated approach, but the absence of a rigorously defined listing of the variables, combined with the possibility of single groups to follow independent lines of research with accessible instrumentation would rather encourage the promotion of several investigators-driven projects. 

If the pursuit of God’s particle, under the species of Higgs’ boson, as approved and enacted by the scientific community, needs a 27-km long, 5 billion €, 10.000 researchers LHC at CERN, Geneva, the Holy Grail of human genetics, *i. e. *the deciphering of our phenotype(s) from the spelling of the A, C, G and T of our genome(s), does not require a similar deployment of investigative weapons. Even assuming it can be found at all, it is possible that similarly relevant advancements would be secured thanks to less structured small scale studies, with minor overall risks if something goes out of control, experimentally as well as conceptually.
